# Morphometric characterization of brain arteriovenous malformations for clinical and radiological studies to identify silent intralesional microhemorrhages 

**DOI:** 10.5414/NP300937

**Published:** 2016-04-06

**Authors:** Melike Pekmezci, Jeffrey Nelson, Hua Su, Christopher Hess, Michael T. Lawton, Melda Sonmez, William L. Young†, Helen Kim, Tarik Tihan

**Affiliations:** 1 Department of Pathology,; 2 Center for Cerebrovascular Research,; 3 Department of Anesthesiology,; 4 Department of Radiology and Biomedical Imaging, and; 5 Department of Neurological Surgery, University of California San Francisco, San Francisco, CA, USA; †Deceased

**Keywords:** arteriovenous malformation, AVM, cerebrovascular disease, hemorrhage, silent intralesional microhemorrhage, stroke

## Abstract

Abstract. Brain arteriovenous malformations (bAVMs) are vascular lesions that can cause significant morbidity and mortality, particularly when they bleed, i.e., rupture. Determining the risk of rupture for bAVMs is a crucial task to determine the most appropriate approach to patients with bAVM. Furthermore, patients who present with a hemorrhagic event also have a higher risk of subsequent hemorrhage. Determination of the hemorrhage risk and management strategy for incidentally discovered bAVMs still remains a controversial subject. In recent years, we have identified silent intralesional microhemorrhages (SIMs) as a possible risk factor for subsequent hemorrhage in patients with bAVMs. The principal aim of this study was to determine critical histological features that can be correlated with preoperative radioimaging findings, and allow better identification of patients with greater risk of adverse outcome. Here we provide a detailed descriptive analysis of the morphometric assessment of bAVMs in order to provide reproducible methodology that will aid in correlating preoperative radioimaging findings with histological features that may be significantly associated with increased risk of hemorrhage/rupture.

## Introduction 

Brain arteriovenous malformations (bAVMs) are considered to be congenital defects of cerebrovascular tree that can be found anywhere, but are most common in the supratentorial region [1, 2]. The original observations highlighted “arterialized” vessels with disrupted elastic lamina, marked vascular mural collagenization, focal proliferation of the smooth muscle layer of the arteries, and inflammatory cells such as macrophages and lymphocytes [3, 4, 5, 6]. Interposed parenchyma contains occasional reactive glial cells, and rarely, hemosiderin pigment. In some cases, no reactive changes are seen. The core of the bAVMs is characterized by a tangle of abnormal arteries and veins, and referred to as the nidus, which has been difficult to ascertain in histology without surgical correlation. While many AVMs have macrophages and marginating neutrophils, occasional lesions harbor significant amounts of parenchymal or perivascular lymphocytic infiltrates [7]. The prevalence and clinical significance of the above-mentioned morphological features of bAVMs have not been well documented. 

The most common symptoms associated with bAVMs are headaches, seizures, or symptoms associated with intracranial hemorrhage. In recent years, “unruptured” AVMs are increasingly being detected in clinical practice in patients who have no associated symptoms, during imaging for unrelated reasons [8]. One of the most critical issues in bAVMs is the possibility of hemorrhage, and this poses a significant morbidity given that nearly half of the bAVMs present initially with a hemorrhagic picture [9, 10, 11]. The annual hemorrhage rate of bAVMs is reported to be ~ 2 – 4% [12, 13]. In addition, bAVM patients who present with intracranial hemorrhage also have a higher risk of subsequent bleeding [11, 12, 14]. A number of grading systems have been devised for the classification and determination of hemorrhage risk in bAVMs. Regardless of the use of any system, the question of what to do with patients with incidental or asymptomatic bAVMs still remains a controversial subject. Our group has previously suggested that silent intralesional microhemorrhage (SIM) may be a risk factor for rupture in bAVMs, and detection of SIM in tissue can help guide radiological studies to detect the presence of hemosiderin when it is in microscopic quantities [15]. The histological finding of hemosiderin also correlated with preoperative iron-sensitive MR imaging study in an attempt to improve risk stratification for bAVM rupture. Specifically, ferumoxytol-based MRI has been used to determine SIMs within bAVMs [16]. All of these studies are predicated on reproducible morphometric assessment of histological findings. Using histological features as gold standard would allow for more accurate characterization of preoperative imaging findings and their use as a marker for SIM. 

To evaluate the association of hemorrhagic presentation with SIM and other histologic features, we have systematically evaluated features of bAVMs which were surgically treated in the same institution over a period of 20 years and were registered in the University of California, San Francisco Brain Arteriovenous Malformation Study Project (UCSFBAMSP). This study constitutes one of the largest series of bAVMs with histological characterization in the literature and identified histological features associated with SIM. 

## Materials and methods 

### Patients 

We identified all patients with surgically treated bAVMs at the University of California, San Francisco (UCSF) between 1989 and 2014 through a database search of the Department of Pathology electronic archive system. Appropriate permissions were obtained from the UCSF Committee for Human Research. The cases identified from the pathology database were than matched with the patients enrolled in the UCSFBAMSP. All available pathology slides and blocks were retrieved and reviewed by two of the authors (TT, MP) who were blinded to the clinical information. Patients with unavailable tissue as well as the specimens that were not diagnosed as AVM or did not have a nidus on histological review were excluded from the histologic evaluation. The patients with familial or vascular syndromes (e.g., hereditary hemorrhagic telangiectasia) were also excluded from the analysis. 

### Histological features 

**Figure 1.**
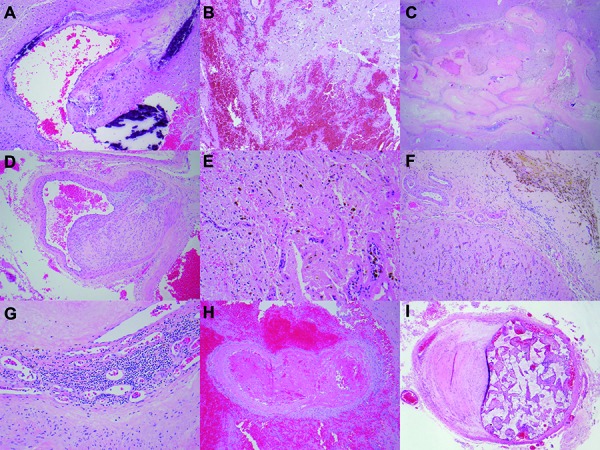
Histological features of brain arteriovenous malformations. A: Calcifications, including mural calcifications; B: Extravasated erythrocytes; C: Dense fibrosis; D: Smooth muscle proliferation; E: Macrophage infiltrate; F: Hemosiderin deposition; G: Lymphocytic infiltrates; H: Fibrin thrombus; I: Intravascular embolization material.

All relevant histological features were recorded on an ordinal scale. A 5-point scale was constructed in terms of quartiles and the number of quartiles in which the feature was present was recorded as outlined below. A graded coverslip was utilized to determine the approximate extent of each histological feature. The number of grids with the histological feature and the total number of grids in the resection material was recorded for each slide. The scoring was considered as “none” or “0” if the feature was absent (0%), “minimal” or “1” if present in 1 quartile of the grids (< 25%), “focal” or “2” if present in 2 quartiles (25 – 50%), “marked” or “3” if present in 3 quartiles (50 – 75%) and “extensive” or “4” if present in all quartiles (> 75%). Each slide was reviewed for the amount of calcifications, extravasated erythrocytes (as defined by clusters of extravasated red blood cells within the parenchyma), dense fibrosis (as defined by increased fibroblastic proliferation and collagenization of the intervening tissues), smooth muscle proliferation (as defined by multilayer proliferation of smooth muscle cells within the vessel walls), the extent of hemosiderin deposition, and the extent of macrophage and lymphocytic infiltrates in the lesion. Figure 1 represents the type of histological features evaluated in the study. Presence of fibrin thrombi within vessels except for those with embolization material, presence of embolization material, presence of intervening brain parenchyma, and presence of arteries within the lesion were recorded as either yes or no. Samples without intervening parenchyma or without arteries were considered not to include the nidus and were excluded from the analysis as a quality control (QC) measure. 

### Statistical methods 

We calculated the frequency and distribution of histological descriptors (hemosiderin, calcification, dense fibrosis, extravasated erythrocytes, lymphocytic infiltrate, macrophage infiltrate, and smooth muscle proliferation) on the aforementioned 5-point scale or, for fibrin thrombi, as present or absent. To measure the level of dependency between the histological descriptors, we performed a non-parametric correlation analysis on each possible pairing, noting Spearman’s rho (ρ). Samples from patients with complete demographic, clinical, and bAVM angioarchitectural (radiological) information were included in a second set of analyses. Summary statistics for this sample subset were calculated both overall and stratified according to whether the patient ever experienced a clinically observed rupture prior to resection. We calculated frequencies of hemosiderin positivity (0 vs. 1 – 4), female sex, Caucasian race, exclusively deep venous drainage, deep AVM location, and associated aneurysm, as well as the mean and standard deviation of age at resection, and maximal AVM size in cm. 

We performed univariable and multivariable logistic regression analyses with any clinical rupture as the outcome to determine whether we could replicate our previous findings reported by Guo et al. [15] that hemosiderin positivity is associated with clinical rupture; results are expressed as odds ratios (OR) and 95% confidence intervals (CI). In an analysis restricted to clinically ruptured patients, we tested whether hemosiderin positivity was associated with the time between the patient’s first rupture and their resection. For this analysis, we used a univariable logistic regression model with hemosiderin positivity as the outcome and the logarithmic elapsed time as the predictor. We performed a similar analysis with lymphocytic infiltrate positivity as the outcome. 

We considered p-values of < 0.05 to be statistically significant. For the 28 pairings in the correlation analyses, we adjusted the significance threshold by using a Bonferroni-corrected significance level of 0.05/28 = 0.002. All statistical analyses were performed using Stata/SE 13.1 [17]. 

## Results 

The initial pathology database search identified 570 cases that were originally diagnosed with AVM. Among those, 504 patients were registered to UCSFBAMSP and were further analyzed. 16 other patients were excluded either because they were not AVMs or were familial cases. 40 of the remaining 488 samples (8.2%) lacked incorporated brain tissue or arteries and failed the quality control parameters, leaving 448 samples in the analysis dataset. Pertinent histological features for these patients are presented in Table 1. 

Hemosiderin was seen in about half of the samples (48.2%). Extensive hemosiderin was seen in 10 (2.2%) samples. Clinical data was available for 7 of these 10 samples, and all 7 came from patients with clear clinical evidence of prior hemorrhage. Fibrin thrombi were present in 53 (11.8%) samples. Pairwise correlations of neuropathology descriptors are summarized in Table 2. Hemosiderin levels strongly and positively correlated with macrophage infiltrate levels (ρ = +0.790, p < 0.001). Hemosiderin was also positively associated with lymphocytic infiltrate (ρ = +0.411, p < 0.001) and dense fibrosis (ρ = +0.216, p < 0.001). 

Complete demographic, clinical, and AVM angioarchitectural radiological information was available for 340 of the 448 patients (75.9%). Summary statistics for this subset of patients are presented in Table 3. We found that 168 (49.4%) patients had clinical evidence of rupture prior to resection. Most of the clinically ruptured cases presented with signs and symptoms of hemorrhage (89.2%). Hemosiderin positivity was associated with clinical rupture in both the univariable (OR = 2.69; 95% CI: 1.73, 4.18; p < 0.001) and multivariable (OR = 2.70; 95% CI: 1.69, 4.34; p < 0.001) models (Table 4). Additionally, exclusively deep venous drainage was associated with clinical rupture in both univariable (p < 0.001) and multivariable models (p = 0.001). Smaller AVMs were associated with clinical hemorrhage in both univariable (p = 0.015) and multivariable analysis (p = 0.011). Associated aneurysms were associated with clinical hemorrhage in the multivariable model (p = 0.019). Deep AVM location was a significant univariable predictor (p = 0.025), but did not remain so in the multivariable model (p = 0.282). Since 71 of the samples in our analysis were included in the original report by Guo et al. [15], we performed a sensitivity analysis rerunning the multivariable logistic regression with only new patients. Again, we found that hemosiderin positivity was positively associated with clinical rupture (OR = 3.15; 95% CI: 1.83, 5.43; p < 0.001). 

**Figure 2.**
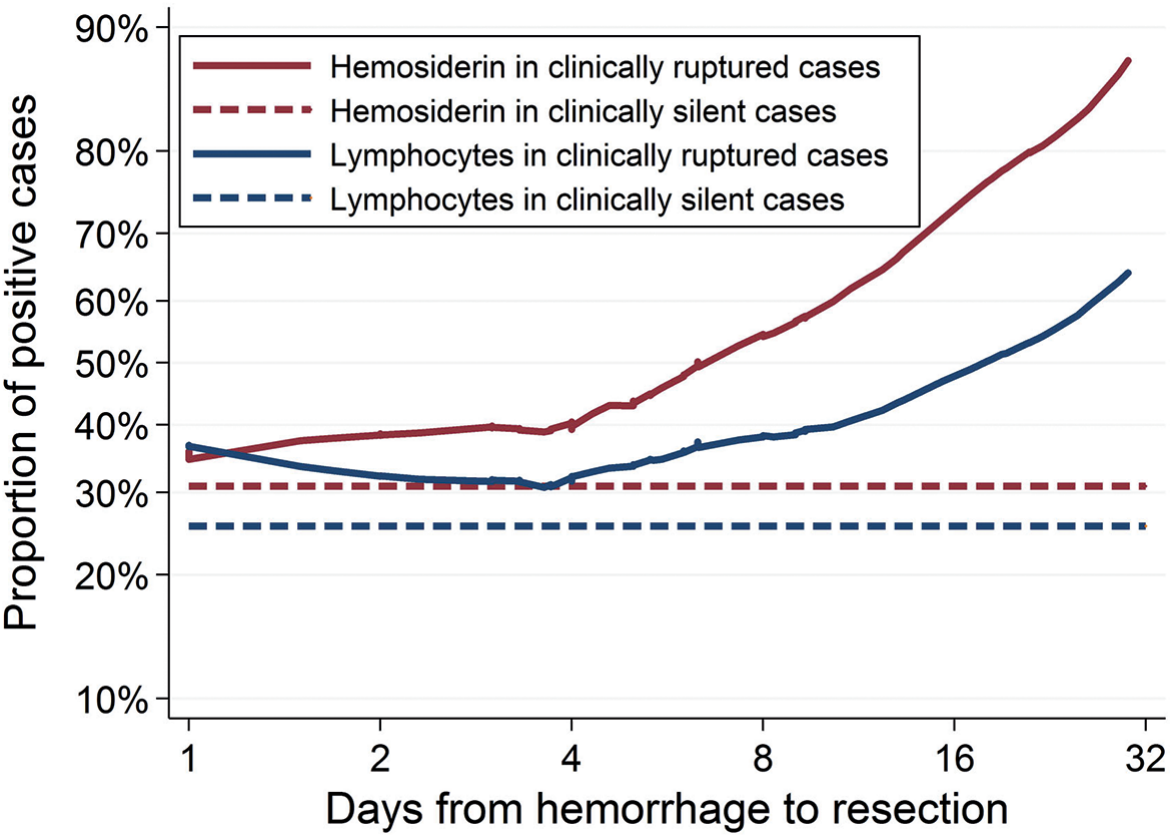
Association between hemosiderin deposition or lymphocytic infiltrate and days from hemorrhagic event to resection. The evaluation of hemosiderin deposition and lymphocytic infiltrates were considered as either yes (any degree from level 1 to 4) or no. “Hemorrhagic samples” refers to patients who had a clinically evident hemorrhagic event. Proportion of cases with hemosiderin deposition is correlated with the elapsed time between hemorrhagic event and the surgical resection (OR = 1.46; 95% CI: 1.06 – 2.00; p = 0.019). Similarly, proportion of cases with lymphocytic infiltrate is correlated with elapsed time between hemorrhagic event and the resection (OR = 1.41; 95% CI: 1.02 – 1.94; p = 0.039).

We found that the amount of elapsed time between clinical evidence of rupture and AVM resection to be associated with increased hemosiderin positivity (Figure 2). When analyzing 104 patients who were operated on within 32 days of clinical evidence of rupture, we found that increasing number of days (specifically each doubling of days) between rupture and resection increased the odds of the sample being hemosiderin positive (OR = 1.46; 95% CI: 1.06, 2.00; p = 0.019). The association between elapsed time and lymphocytic infiltrate was also significant (OR = 1.41; 95% 1.02, 1.94; p = 0.039). 

## Discussion 

The main goal of this manuscript is to present the morphometric methodology that provides the fundamental studies on bAVMs and provide an objective comparison of our results with prior and subsequent studies. While the analyses of histological findings further establish the morphological evidence of pathological processes in bAVMs, they also point to some interesting features that require further study or inquiry. This study further supports our initial assertions that histological identification of hemosiderin within the tissues of bAVMs is indeed associated with clinical hemorrhage, and it may be possible to utilize this parameter to identify alternative modalities to assess SIM and its role as an independent risk factor. 

Presence of hemosiderin in bAVMs has often been considered unusual as opposed to cavernous angiomas (cavernous malformations) that are distinctly different pathological lesions and harbor a ring of hemosiderin that is often recognizable radiologically [18]. Nevertheless, prior histological studies have noted rare hemosiderin deposits in some bAVMs, often associated with hemorrhage [19]. In our study, we have identified that a hemosiderin score of 4 (extensive) was almost always associated with a history of significant hemorrhagic event. However, lower hemosiderin scores, i.e., minimal and focal hemosiderin presence, were less likely to be found in patients with clinical or radiological evidence of hemorrhage or a hemorrhagic presentation. There can be little or no doubt that the presence of hemosiderin within the parenchyma of bAVMs is an indication of prior leakage through the abnormal vessels in bAVM, given the subsequent oxidation of extravasated hemoglobin to hemosiderin will require time. This mechanism has been well recognized for a long time, and has also been shown in relation to micro-hemorrhages in other conditions [20, 21]. Therefore, the finding of low levels of hemosiderin should be considered as evidence of SIMs. Compared to patients without SIM, patients with SIM seem to have a higher rate of clinical hemorrhage. Therefore, we suggest that demonstration of hemosiderin in tissues of bAVMs is associated with a higher risk of adverse outcomes, and such patients should be more carefully followed and treatment options selected accordingly. However, we note the main limitation of our study that histologic findings can only be assessed in surgically operated cases, which creates a selection bias. The true risk of hemorrhage associated with SIM should be evaluated in prospective clinical trials with diagnosis of SIM via imaging. 

Our data also demonstrate that in cases of known hemorrhagic event, there is a strong correlation between the extent of hemosiderin deposition and the time of hemorrhagic event. This is not surprising given the longer the time gap is, the more likely the blood products of recent hemorrhage have time to be converted to hemosiderin. However; it is critical to note that ~ 1/3 of the cases operated on the day of hemorrhage show hemosiderin accumulation, which cannot be related to the most recent hemorrhagic event. Furthermore, this trend can provide additional information regarding the rate of hemosiderin accumulation when the interval between the hemorrhage and surgery is known. 

We have found strong correlation with the presence of macrophages and hemosiderin deposition. This is quite expected, since the natural response to extravasation and leakage of blood products into the brain parenchyma would be a natural trigger for a macrophage response. The strong correlation between these two parameters suggests that the main reason for the presence of macrophages in bAVMs can be attributed to tissue extravasation and damage. This would naturally suggest that imaging modalities that can track macrophage infiltrates can also be used as surrogate markers of SIMs. While ischemic events and infarcts in association with a bAVM should also be considered as a reason for macrophage infiltrates, this would not be in association with hemosiderin. 

The presence of lymphocytic infiltrates in bAVMs has been a curious finding, and it was not clearly evident to us that perivascular and parenchymal clusters of lymphocytes should be directly associated with extravasation and subsequent inflammatory response. There is evidence to suggest that lymphocytic infiltrates may be associated with inflammatory or immune responses that are not directly related to hemosiderin or macrophage deposition [22]. We have not quantified or characterized the type of lymphocytic infiltrates beyond determining its extent as measured in the 5-tiered scale. Nevertheless, we could not provide additional support that lymphocytic infiltrates in bAVMs could be associated with a distinct immune response or inflammatory event [22]. The time-course analysis of the extent of lymphocytic infiltrates demonstrated a similar trend with hemosiderin in relation to the hemorrhagic event, suggesting that the increase in the lymphocytic infiltrates may be partially attributed to the hemorrhage. However, our inability to demonstrate any other cause does not provide conclusive evidence that the presence of lymphocytic infiltrates can be explained exclusively by the hemorrhagic event. There may still be additional immune mechanisms influencing an inflammatory response to bAVM, potentially leading to the hemorrhagic event. These findings should be taken into consideration in future studies that attempt to further characterize the nature of lymphocytic response and the effect of the immune system in the biology of bAVMs. 

Two common histologic findings, dense fibrosis of the abnormal vessel walls and the proliferation of the smooth muscle cells of bAVM vasculature have been well known but not so well explained. These two features are quite prevalent in many bAVMs, and they seem to correlate positively with each other. Dense fibrosis correlates positively with hemosiderin and macrophage infiltrates. These findings suggest that the mural changes in the vessels of bAVMs may render these vessels prone to leakage and may influence the occurrence of SIMs or even frank hemorrhage. However, how these changes exert their influence is unclear and most likely quite complex. Alternatively, they may represent tissue response to hemorrhage and/or inflammatory infiltrate. This study was not designed to identify a causal association between smooth muscle proliferation or dense fibrosis and SIM. We expect to focus on these features in future studies in our model systems that allow modulation of these parameters [23, 24]. 

In conclusion, we have demonstrated a series of histological features to be present in the majority of bAVMs, some of which still require characterization in terms of their contribution to biology of these malformations. Presence of macrophages and hemosiderin is seen in a significant number of bAVMs, and their value in determining the existence of SIM as a risk factor for subsequent hemorrhage needs to be further investigated. Our attempts to characterize the histological findings in bAVMs will hopefully help in preoperative imaging characterization of these lesions, and further determine the value of early intervention in selective cases. The significance of histological features such as dense fibrosis, smooth muscle proliferation, and lymphocytic infiltrates also need to be better determined in prospective studies. 

## Conflict of interest 

None of the authors have any conflict of interest, financial or otherwise, related to the contents of this manuscript. This work was supported by an R01 grant (NS034949) from the National Institutes of Health. 

**Table 1.** Distributions of histological descriptor levels from 448 AVM samples.

Characteristic	None	Minimal	Focal	Marked	Extensive
	0%	< 25%	25 – 50%	50 – 75%	> 75%
Hemosiderin	232 (52%)	80 (18%)	84 (19%)	42 (9%)	10 (2%)
Macrophage infiltrate	189 (42%)	115 (26%)	88 (20%)	40 (9%)	16 (4%)
Calcification	369 (82%)	46 (10%)	21 (5%)	10 (2%)	2 (< 1%)
Dense fibrosis	11 (2%)	111 (25%)	255 (57%)	64 (14%)	7 (2%)
Extravasated erythrocytes	57 (13%)	144 (32%)	152 (34%)	69 (15%)	26 (6%)
Lymphocytic infiltrate	272 (61%)	131 (29%)	39 (9%)	6 (1%)	0 (0%)
Smooth muscle proliferation	20 (4%)	205 (46%)	195 (44%)	28 (6%)	0 (0%)

Entries are presented as the number of cases and percentage (%). Presence of fibrin thrombi is scored as present or absent and is present in 53 (12%) of cases.

**Table 2.** Non-parametric correlation of histological descriptors on the 5-point scale.

	Ca	DF	EE	FT	He	LI	MI	SM
Ca	1							
						
DF	0.267	1						
	***< 0.001***							
EE	–0.006	0.022	1					
	*0.900*	*0.651*						
FT	0.144	0.066	0.220	1				
	***0.002***	0.161	***< 0.001***					
He	0.051	0.216	0.140	0.031	1			
	*0.278*	***< 0.001***	*0.003*	*0.515*				
LI	0.097	0.114	0.250	0.110	0.411	1		
	*0.040*	0.016	***< 0.001***	*0.020*	***< 0.001***			
MI	0.065	0.176	0.319	0.152	0.790	0.570	1	
	*0.168*	***< 0.001***	***< 0.001***	***0.001***	***< 0.001***	***< 0.001***		
SM	0.079	0.366	–0.040	–0.051	–0.050	–0.011	–0.045	1
	*0.093*	***< 0.001***	*0.403*	*0.278*	*0.296*	*0.824*	*0.338*	

Ca = calcification; DF = dense fibrosis; EE = extravasted erythrocytes; FT = fibrin thrombi; He = hemosiderin; LI = lymphocytic infiltrate; MI = macrophage infiltrate; SM = smooth muscle proliferation. Presence of fibrin thrombi is scored as present or absent and not on the 5-point scale. All patients with complete pathology information are included in this table (N = 448). The first number in each box is Spearman’s ρ-value, and the lower numbers are p-values. Statistically significant p-values are highlighted in bold.

**Table 3.** Demographic and clinical characteristics of 340 AVM patients with complete clinical information.

	Ever-hemorrhagic		
	No (n = 172)	Yes (n = 168)	Overall (n = 340)
Hemosiderin present	57 (33%)	96 (57%)	153 (45%)
Age at resection (years)	37.2 ± 14.9	35.4 ± 18.2	36.1 ± 16.6
Female sex	92 (53%)	87 (52%)	179 (53%)
Caucasian	95 (55%)	70 (42%)	165 (49%)
Rupture at presentation	0 (0%)	150 (89%)	150 (44%)
Exclusively deep venous drainage	8 (5%)	37 (22%)	45 (13%)
AVM size (cm)	2.9 ± 1.2	2.6 ± 1.3	2.7 ± 1.2
Deep location	13 (8%)	26 (15%)	39 (11%)
Associated aneurysm	47 (27%)	61 (36%)	108 (32%)

Table entries are total number of cases with the attribute. The numbers in parentheses are percent values. Age at resection in years and AVM size in centimeters are given as mean ± standard deviation. Ever-hemorrhagic implies any clinical evidence of hemorrhage at any given time during the follow-up time.

**Table 4.** Odds ratios for clinical and histological features as determinants of clinical hemorrhagic events prior to resection (N = 340).

	Univariate analysis			Multivariate analysis		
	OR	95% CI	p-value	OR	95% CI	p-value
Hemosiderin positive	2.69	(1.73, 4.18)	< 0.001	2.70	(1.69, 4.34)	**< 0.001**
Age at diagnosis (decade)	0.94	(0.82, 1.06)	0.309	0.92	(0.79, 1.06)	0.248
Female sex	0.93	(0.61, 1.43)	0.753	0.78	(0.48, 1.25)	0.302
Caucasian	0.58	(0.38, 0.89)	0.013	0.57	(0.35, 0.91)	**0.018**
Exclusively deep venous drainage	5.79	(2.61, 12.86)	<0.001	4.65	(1.94, 11.15)	**0.001**
AVM size (cm)	0.80	(0.67, 0.96)	0.015	0.76	(0.62, 0.94)	**0.011**
Deep location	2.24	(1.11, 4.52)	0.025	1.61	(0.68, 3.81)	0.282
Associated aneurysm	1.52	(0.96, 2.40)	0.076	1.86	(1.11, 3.13)	**0.019**

OR = odds ratio; CI = confidence intervals.
